# Auditory chaos classification in real-world environments

**DOI:** 10.3389/fdgth.2023.1261057

**Published:** 2023-12-21

**Authors:** Priyanka Khante, Edison Thomaz, Kaya de Barbaro

**Affiliations:** ^1^ Department of Electrical and Computer Engineering, The University of Texas at Austin, Austin, TX, United States; ^2^ Department of Psychology, The University of Texas at Austin, Austin, TX, United States

**Keywords:** auditory classification, deep learning, household chaos, real-world dataset, developmental psychology

## Abstract

**Background & motivation:**

Household chaos is an established risk factor for child development. However, current methods for measuring household chaos rely on parent surveys, meaning existing research efforts cannot disentangle potentially dynamic bidirectional relations between high chaos environments and child behavior problems.

**Proposed approach:**

We train and make publicly available a classifier to provide objective, high-resolution predictions of household chaos from real-world child-worn audio recordings. To do so, we collect and annotate a novel dataset of ground-truth auditory chaos labels compiled from over 411 h of daylong recordings collected via audio recorders worn by N=22 infants in their homes. We leverage an existing sound event classifier to identify candidate high chaos segments, increasing annotation efficiency 8.32× relative to random sampling.

**Result:**

Our best-performing model successfully classifies four levels of real-world household auditory chaos with a macro F1 score of 0.701 (Precision: 0.705, Recall: 0.702) and a weighted F1 score of 0.679 (Precision: 0.685, Recall: 0.680).

**Significance:**

In future work, high-resolution objective chaos predictions from our model can be leveraged for basic science and intervention, including testing theorized mechanisms by which chaos affects children’s cognition and behavior. Additionally, to facilitate further model development we make publicly available the first and largest balanced annotated audio dataset of real-world household chaos.

## 1. Introduction

Household chaos—characterized by an environment high in noise and crowding and low in regularity and routines (1)—is an established risk factor for child development, affecting both brain and behavior development (2, 3). Households that have high levels of chaos are associated with increased child behavior problems, including decreased self-regulation, attention and arousal, and increased levels of aggression (2–4), each associated with increased risks for child disruptive behavior disorders such as oppositional defiant disorder and conduct disorder (5). Higher household chaos is also linked to worse child cognitive performance, including lower IQ (3), lower academic achievement (6) and poorer reading and language skills (7, 8). Finally, chaotic households also are associated with harsher and less sensitive parenting practices (9–11) which can both lead to and reinforce maladaptive trajectories of child development. Thus, objective, accessible, remote measures of household chaos could be part of a preventative approach for identifying and mitigating child development and behavior problems.

Research in developmental science typically measures chaos using surveys completed by caregivers living in the home (2, 3). However, these measures are subjective, meaning that caregivers with different personalities or perceptions may have different thresholds for making chaos judgements. Objective markers of chaos, for example, markers automatically detected from audio recordings, would allow for more systematic assessments of this risk factor. Additionally, current survey methods provide static measures of chaos, reflecting a caregiver’s overall assessment of the chaos in their home. However, household chaos is likely a dynamic feature of an environment with dynamic effects on children’s behavior. Once mobile, children play an active role in determining their sensory inputs in real time (12, 13). For example, a highly reactive child may be more likely to seek out spaces in the home that are quieter and less stimulating. Alternatively, a highly surgent or ebullient child may seek stimulation and indeed create it. Dynamic objective measures of auditory chaos in real-world household settings would allow researchers to develop and test more specific mechanisms by which chaos is hypothesized to affect child outcomes. This is critical in that much of the prior work cannot disentangle to what extent high chaos environments are a cause or consequence of child behavior problems. For example, the temperamental factor of child surgency is also a risk factor for later externalizing behaviors (14). Thus, the association between household chaos and externalizing disorders could be in part driven by the fact that more surgent children are likely to contribute to increased levels of household chaos. Dynamic measures of household auditory chaos could be used to disentangle and clarify such complex possibilities. For example, by examining real-time sequences of hypothesized predictors and consequences of chaos in real-world scenarios, researchers could test bidirectional influences between chaos and physiological arousal, focused attention, or sleep (15–17), and whether characteristics such as child temperament moderate these relationships. However, there are no available models to detect household chaos from auditory recordings collected in children’s everyday environments.

A growing community of developmental scientists and engineers are collaborating to develop algorithms to detect and classify developmentally relevant activities from sensors worn by children in natural everyday environments (18–20). These include models that can detect parent and child sensory inputs, emotions, behaviors, and contexts in order to understand learning and development in everyday settings (21–25). Detected behaviors have also been leveraged for early childhood interventions (26–29). In this paper, we contribute to this broader effort by developing a multi-class classifier for *auditory chaos* using daylong audio recordings collected by an infant-worn audio sensor.

The major contribution of our paper is to build a multi-class auditory chaos classifier that classifies input audio segments into four levels of chaos. We define these classes based on descriptions of chaotic environments in the developmental psychology literature, specifically, using the gold-standard questionnaire measures that are most commonly used to assess household chaos (30, 31). Periods of silence and sounds that are low in volume or contain only a single source of sound are classified as relatively low auditory chaos (Chaos 0 or 1, respectively). Time periods with sounds that are high in volume, potentially jarring, or cacophonous in nature are classified as high in auditory chaos (Chaos 3). Table 1 provides additional examples and description of our four-level auditory chaos spectrum, along with some examples on the types of everyday sounds included in each category. From an engineering perspective, this problem is distinct from typical auditory classification tasks in that the task here is to classify the quality of an environment in terms of relative degrees of auditory stimulation rather than identifying distinguishing characteristics between specific sounds or groups of sounds, as is the case for traditional sound event or acoustic scene classification tasks, respectively. Therefore, chaos classification poses a modeling challenge insofar as the model needs to go beyond learning individual sounds or groups of sounds, instead learning high level representations of the overall soundscape, including the proportion of overlapping sounds, number of sound sources, or the jarring or cacophonous nature of sounds contained in an audio recording.

**Table 1:** Definitions and examples of types of sounds for the four levels of auditory chaos. Note that the list of examples provided is not exhaustive.

Chaos levels	Definition	Examples of types of sounds	Examples of YAMNet classes
No chaos (0)	Silence or absence of sounds	—	Silence, Pulse, Heart sounds/Heartbeat, Breathing
Low chaos (1)	Soft daily or familiar sounds	Conversation between parents at normal volume, low volume calming music, distant wind chimes, walking, stroller on gravel, pouring water, low dishwasher/microwave hum, white noise machine	Wind, Singing, Chime, Classical music, Piano, Raindrop, White noise, Shuffling cards, Tearing, Drip, Purr, Microwave, Walk/footsteps
Medium chaos (2)	Slightly stimulating sounds	Commanding/raised voices, loud singing, baby laughing, another child playing/running around, low-volume TV, toy music, rattle, shower, faucet, blow dryer, vacuum cleaner	Child speech/kid speaking, Toilet flush, Electric shaver, Doorbell, Alarm clock, Hair dryer, Pop music, Acoustic guitar, Violin/fiddle, Sink (filling or washing)
High chaos (3)	Highly stimulating, scary, or jarring sounds	Adults arguing, shouting, many children playing, baby wailing, loud TV, crashing sounds, loud dog barking, crows cawing, restaurants, swimming pools, cars honking, drums, trumpets, blender	Children shouting, Screaming, Car, Traffic noise/roadway noise, Applause, Drum roll, Electronic music, Fire alarm, Tools, Chain-saw, Drill, Inside/public space, Battle cry

In our aim to build a multi-class auditory chaos classifier, we make the following contributions:

• We construct and evaluate a high chaos detector to efficiently annotate data to train and test our classifier. Our detector improves annotation efficiency of rare *high chaos* events by a factor of 8.32, allowing us to annotate only 9.85% of 244.3 h of raw daylong recordings and providing us with 4h of ground truth high chaos data for model development.
• We develop and compare multiple real-world auditory chaos classification models. Our best-performing model achieves a macro F1 score of 0.701 (Precision: 0.705, Recall: 0.702) and a weighted F1 score of 0.679 (Precision: 0.685, Recall: 0.680) across all four levels of chaos.
• Using a data ablation study, we determine the benefit of a large training dataset (∼55 h) for model performance. By varying the amount of training data, we find that the model’s macro and weighted F1 score increases by 4.0% and 4.6% respectively, when the amount of training data increases from 5h to 40h.
• We make a subsample (39.4 h) of our human annotated auditory chaos dataset publicly available,^1^ representing the largest and the only dataset of auditory chaos currently available. This subsample includes all audio data from only those participants that consented to share their data with other researchers; the rest of it remains private. We also make our best-performing auditory chaos multi-class classifier publicly available^2^ for research applications.

## 2. Related works

This study is a pioneer effort to build an auditory chaos multi-class classifier, so there is no known benchmark for comparison. However, in this section, we discuss the traditional approaches used in developmental psychology to measure household chaos and highlight how our current work differs from the previous efforts, highlighting the value added of our work. Additionally, we present relevant works in the domain of auditory classification and in the creation of large annotated datasets. These works inspired our modeling approach and the development of the high chaos detector, a tool that we leveraged to construct our large auditory chaos labeled dataset.

### 2.1. Measuring household chaos

Household chaos, characterized by noise, disorganization, and lack of routines in the home, has been associated with adverse outcomes for both children and caregivers. In the developmental community, household chaos has typically been measured through the Confusion, Hubbub and Order Scale (CHAOS) a subjective survey completed by the caregiver (30). Some work is based on trained observers making detailed observations of participant’s homes through Descriptive In-Home Survey of Chaos—Observer ReporteD (DISCORD) (31). Thus, most previous research on household chaos (32–35) has relied upon static or invariant measures that correspond to either an “overall” level of chaos in the household, as perceived by the caregiver, or a single snapshot of household chaos.

One recent publication (36) used volume of infant-worn audio recordings as a minute-by-minute dynamic measure of household chaos. However, our preliminary analyses and baseline models suggest that volume is not a robust measure of household chaos (see Section 5.1.1, Section 6.1). For example, an adult gently speaking to an infant at close proximity may have a greater volume and amplitude than a TV playing in the background. In such situations, volume would provide erroneous measures of household chaos. More broadly, as volume is directly proportional to the distance from the audio sensor, volume alone is not a good measure of chaos.

As such, we propose to train a real-world auditory chaos classifier grounded in the existing developmental psychology literature on chaos (30). Our classification of chaos is drawn from the gold-standard CHAOS survey items relating to the auditory components of household chaos. For example, items including “You can’t hear yourself think in our home,” “I often get drawn into other people’s arguments at home” and “The telephone and the TV take up a lot of our time at home” were used as the basis of our annotation scheme. Given the fact that these questions are responded to by a caregiver living in the home, we infer that auditory household chaos should include sounds made by the target infant, children, and other family members in the home.

### 2.2. Audio classification

We know of no existing models that aim to classify auditory chaos. To gain insights into developing a model for auditory chaos classification, we review recent work in sound event and acoustic scene classification—two domains most related to chaos classification. The auditory signal processing and ubiquitous computing communities have made strong gains in audio event detection and scene classification. Prior works in the field of audio classification span a range of tasks. Many past works do binary classification of specific individual sounds including coughing, laughing, snoring, screaming, or infant crying (22, 37–40). Other efforts have explored multi-class classification, including classifying multiple individual types or categories of sounds (41–44), for example, animal, natural soundscapes and water sounds, human speech and non-speech sounds, domestic, urban and source-ambiguous sounds. These efforts typically leverage publicly available datasets including e.g. ESC-10 and ESC-50 (45), UrbanSound (46), CHiME-home (47) and Audio Set (48). Other multi-class classification efforts have focused on classifying groups or combinations of sounds in the form of scenes (49, 50), for example, training models to detect that *dishes clanking, water tap running, and cupboard* sounds typically occur in a *home* environment, or that *car horn, vehicle sounds, and breeze* most likely indicate an *busy street* environment. Multi-class sound and acoustic scene classification are relevant to auditory chaos classification insofar as chaos classification also requires the model to learn representations of multiple sounds or groups of sounds in the environment to determine the chaos level of that environment.

Many of these works have achieved good or very good performance on multi-class classification, indicating that models can learn distinguishing acoustic features between individual sounds or groups of sounds. Early models used traditional machine learning techniques such as Support Vector Machines, Gaussian Mixture Models and K-Nearest Neighbours with extracted acoustic input features including mel-frequency cepstrum coefficients (MFCC), temporal, spectral, energy and prosodic features (51–55). However, currently, most state-of-the-art models use deep learning techniques to classify sound events or scenes (56–58). Given large amounts of data, deep learning models can extract complex high-level features that can better distinguish between sounds and scenes rather than the pre-selected typically low-level features provided to traditional machine learning algorithms. In the current paper, we test out both—traditional machine learning and deep learning approach—to auditory chaos model development as there is no previously established baseline for the task of auditory chaos classification. As auditory chaos classification is a complex task where distinguishing between chaos levels depends not only on low-level acoustic features such as MFCCs, loudness and energy but also on high-level features such as proportion of overlapping sounds, level of “cacophony,” etc., we hypothesize that the deep learning approach might perform the best.

A key consideration for model application is whether models are trained and tested on real world data. Models constructed with data collected in “clean” laboratory environments have a high performance on those datasets, but do not generalize to real-world settings (22, 37, 38, 59, 60). Real-world data is more unstructured and noisy than lab-based data, and typically contains a more variable examplars of sound classes. Therefore, real-world data is generally thought to pose a harder challenge for models to learn from and maintain consistent performance. As the ultimate goal of our auditory chaos model is to understand the dynamic effects of chaos on child development as it occurs in children’s everyday environments, it is essential that our model works in real-world settings. We, therefore, undertake the task of real-world auditory chaos classification.

Auditory chaos classification is different from these aforementioned audio event and acoustic scene classification works, but can likely draw from them. Similar to acoustic scene classification, chaos classification depends upon considering groups of sounds rather than identifying specific sounds. However, the goal is to distinguish the quality of different environments rather than sounds that can be used to distinguish different types environments from one another. This is challenging in that two highly chaotic instances of real-world audio might not have any overlap between the characteristic sound qualities that classifies them as highly chaotic. For example, an audio segment could be classified as having a high (level 3) level of chaos due to the presence of a single loud sound, such as of a loud bang or dog barking, or a cacophony of quieter sounds occurring over time, such as in a crowded restaurant. Moreover, a given sound class can be highly chaotic in one instance but not in another depending on its characteristics in that instant. For example, the class *speech* can be highly chaotic if a person is shouting or screaming but not chaotic when gently speaking to an infant. Thus, the chaos classifier must learn a high level representation that goes beyond the individual sounds or even types of sounds.

### 2.3. Annotation of rare events

A supervised approach for auditory chaos classification requires an annotated dataset to train and test the classifier. However, creating a large enough dataset to build a successful model for auditory chaos classification is challenging as instances of high chaos are relatively rare in everyday life. For example, annotation of 14.1 h of our raw audio recordings led to highly imbalanced annotated dataset with only 1.02 h of high chaos (Chaos 3). This feature is not unique to high chaos alone; other everyday sounds, such as coughing or infant crying also occur rarely during daylong recordings. To get enough ground truth to train and test their models, some previous works have annotated large volumes of audio data e.g. (22, 37). One strategy for annotating large volumes of audio data is to outsource annotation via crowdsourcing, which was employed to create Audio Set (48) and OpenMIC-2018 (61). However, crowdsourcing can fall short for annotation tasks that require domain expertise. Additionally, for many datasets collected by the developmental science community, incuding first-person wearable audio datasets such as our own, crowdsourcing could violate participant privacy and is therefore often not an option. Moreover, issues have been raised about the quality of annotations collected (62–64) as the primary motivation of the online workers tend to be monetary (65).

Another domain dealing with the challenge of rare events is the field of auditory anomaly detection. It is hard to collect data for anomalies or abnormal events such as gunshots, screams, glass breaking and explosions in the real world as their occurrences are quite rare. To circumvent this problem, to obtain enough data to develop classification models for abnormal events, previous works have leveraged artificially curated datasets created by superimposing the rare events on background noises from different environments (66–68). Others have collected data by having actors create and enact abnormal situations (40). However, such artificially constructed datasets and data collected in structured laboratory contexts do not reflect real-world settings, and hence do not generalize to the real-world scenarios that they are intended to function in (22, 37, 38, 59, 60).

We therefore undertake the task of collecting and annotating real-world audio recordings to ensure that our auditory chaos classifier works in real-world settings. Inspired by works dealing with annotation of large real-world audio recordings (22, 48, 61, 37), we take the approach of identifying candidate sets for the rare high chaos class for annotation instead of annotating the entire dataset. Candidate sets represents a set of audio segments that have a high likelihood of containing the class of interest. Audio Set, a large-scale audio dataset containing 632 labeled sound events, (48) followed a multi-modal approach to select candidate sets prior to annotation via crowdsourcing. This included leveraging other sources of information like metadata, anchor text and user comments to predict events in videos. Videos with high scoring predictions were chosen as candidate videos for annotation. Additionally, they used weak search engines to select candidate videos with high confidence.

Other previous works have employed the use of specific classifiers for candidate set selection. For example, OpenMIC-2018, a dataset for multi-instrument classification, trained a classifier on Audio Set classes specific to musical instruments to select candidate audio samples for annotation. Similar approaches have been used to select sound intervals of high likelihood for snoring and infant crying (22, 39, 69).

We draw from these aforementioned works to design a high chaos detector (detailed in Section 4) that also leverages an existing classifier, YAMNet (41), trained on Audio Set (48) to output a candidate set of high chaos audio segments to be annotated. Our annotation task has distinct challenges relative to those undertaken by previous works. In particular, we only have one source of information at our disposal (audio), whereas Audio Set had multiple (metadata, user comments, links, etc.). Next, given that no prior models for auditory chaos classification have been developed, we cannot use a direct one-to-one mapping from existing classifiers. As such, there is a need for a creative solution to map the predicted labels from an existing audio classifier to our four levels of auditory chaos to select candidate segments.

## 3. Modeling and data overview

**Figure 1:**
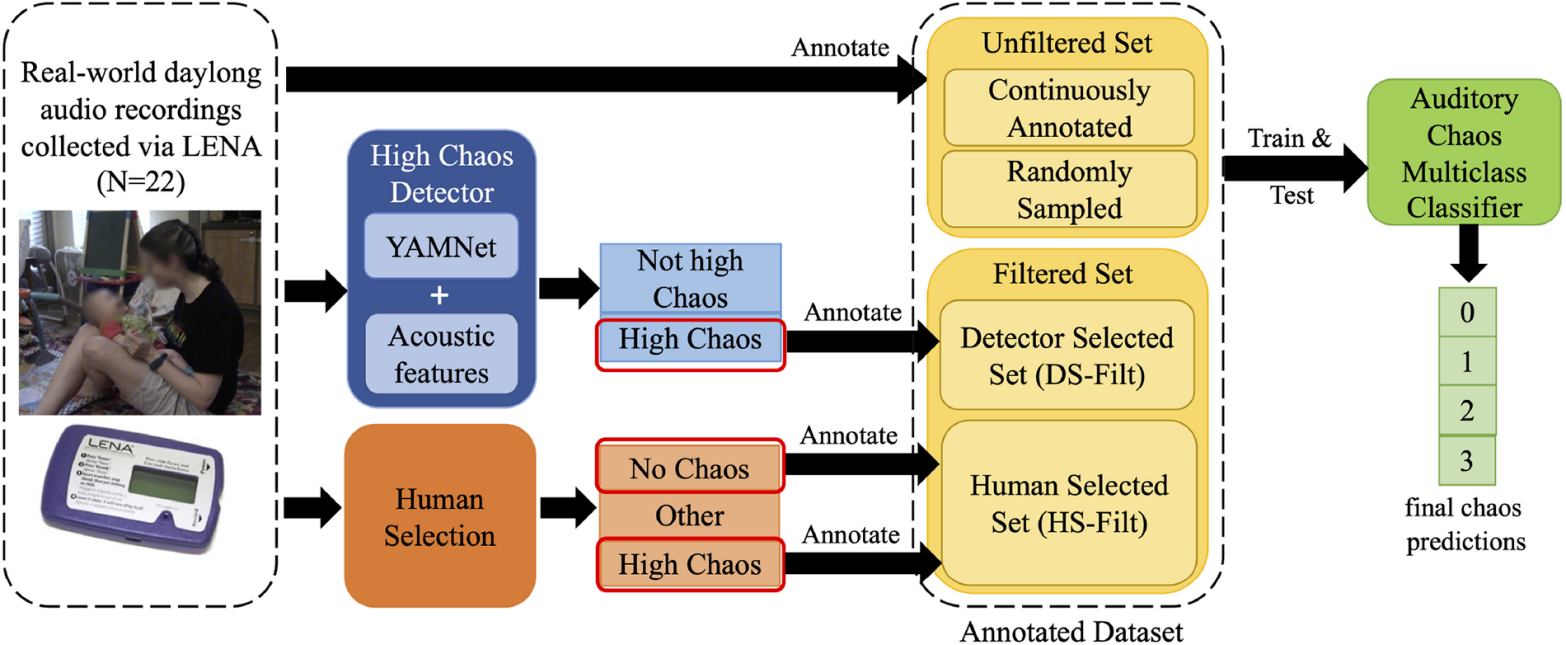
Flowchart of our auditory chaos classification model development.

Here, we outline our process for constructing an auditory chaos classifier, as detailed in subsequent sections of our manuscript and illustrated in Figure 1. First, we collect a dataset from real-world infant-worn audio recordings. Next, to train and test our model we obtain and annotate data via three primary pathways: 1) human annotation of unfiltered data, 2) by developing and using a High Chaos Detector, and 3) human selection of additional candidate segments. Finally, we combine data from these three pathways to form the Annotated dataset, which we use to train and test machine learning models for the real-world auditory chaos classification task, detailed in Section 5.

### 3.1. Device

Our daylong audio recordings are continuously recorded in naturalistic unstructured home settings using the LENA (Language ENvironment Analysis) audio sensor (18) worn by infants in a vest. LENA records all audio occurring within 6–10 feet of the infant and can record continuously up to 24 h on a single charge. All audio is stored in PCM format one 16-bit channel at a 22.05 kHz sampling rate (70).

### 3.2. Participants

88 families enrolled in the broader study and audio data was collected from 78 participants. The broader study aimed at utilizing wearables to characterize mother-infant interactions in everyday home settings (22, 71). All participants lived in a mid-sized urban city. All participants provided informed consent for using the data in subsequent analyses, including the present study. Due to the time-intensive nature of auditory chaos annotation, 22 participants were selected from this larger set of 78 participants for the current study. These 22 participants were selected based on the following criteria: English-speaking families who shared at least one 12+ hour continuous LENA recording. To ensure socioeconomic representation, we selected participants with different levels of income and education. Table 2 depicts sample characteristics.

**Table 2:** Participant characteristics (n=22).

	n (%)	M (SD), range
Mother age, years		30.7 (5.5), 22–43
Infant age, months		5 (6.5), 0.87–33
Infant sex, female	11 (50%)	
Race/Ethnicity		
non-Hispanic White	13 (59.1%)	
Hispanic	5 (22.7%)	
More than one	2 (13.6%)	
Black	1 (4.5%)	
Maternal education		
High school or less	2 (9.1%)	
Some college or trade school	6 (27.3%)	
College	7 (31.8%)	
Graduate school	7 (31.8%)	
Family status		
Married	18 (81.8%)	
Single parent	1 (4.5%)	
Living with a partner without marriage	3 (13.6%)	
Household income		
Under $25k	2 (9.1%)	
$25k–$49k	3 (13.6%)	
$50k–$74k	6 (27.3%)	
$75k–$99k	2 (9.1%)	
Over $100k	9 (40.9%)	
Number of other children in the home		1 (1.3), 0–5

### 3.3. Annotation scheme

To facilitate the training and testing of the auditory chaos classifier, all 411.2 h of data collected from 22 participants were segmented into 296064 non-overlapping continuous 5 s long audio segments. As the primary reason to build this model is to capture the dynamic changes in chaos, having an automated measure that predicts chaos levels at a high granularity is preferable. Additionally, if desired, outputs at a finer granularity can be combined to obtain chaos measures over a larger timescale i.e. a minute or an hour or even a day. As some high chaos events can last only a couple of seconds, for example, a loud bang or a bark, we chose the 5 s timescale to be able to capture these changes. Furthermore, we follow previous works who have used 0.5–5 s audio segments for sound event classification (37, 22, 42) or acoustic scene classification (43, 44), domains most related to our auditory chaos classification task.

A subset from these 296064 segments were annotated by trained research assistants as detailed in Sections 4.2 and 5.2. All annotations were done on a segment-level. Each segment was annotated as one of four levels of auditory chaos, namely, no chaos (0), low chaos (1), medium chaos (2) and high chaos (3), with each segment having only one chaos label. Sample sounds for each chaos level are described in Table 1 and the complete auditory chaos annotation scheme can be found in Supplementary Section 1. Annotators included all sounds made by children and infants (e.g. laughing, yelling, crying), including the target infant wearing the audio recorder in their determination of the chaos levels for a segment. For example, loud infant crying would be labelled as high chaos (level 3). The gold-standard CHAOS questionnaire includes items such as “It is a real zoo in here” and “I can’t hear myself think,” which would certainly include sounds made by infants, children, and other family members in the home. Given that this particular measure of household chaos has been found to be predictive of children and parents’ outcomes in the developmental literature (32–35), it is essential to adhere to this definition of chaos in developing an auditory chaos classification model.

Typically, a segment was annotated with the max chaos level of all the sound classes it contained. However, it is important to note here that the chaos level assigned to a segment did not always depend only on sound classes it contained but was also labeled by taking into consideration the overall cacophonous nature of the segment. This is also consistent with the CHAOS questionnaire items, for example, “There is often a fuss going on at our home” which could refer to multiple ongoing events contributing to high auditory chaos. For example, multiple medium (level 2) chaos sounds happening simultaneously could lead the segment to be marked as high (level 3) chaos even though the max chaos level of all sounds classes is 2. We include our detailed annotation scheme in the Supplementary Section 1. Annotations were conducted according to best practices in behavioral sciences (inter-rater reliability kappa score (72): 0.76, corresponding to strong agreement).

### 3.4. Datasets

To obtain a dataset to train and test our auditory chaos models, we first constructed two separate datasets—the *Unfiltered* set and the *Filtered* set. Table 3 summarizes the volume of data annotated and the number of participants in each set.

**Table 3:** Summary of all annotated data.

Annotated dataset		Participants	Recordings	Hours	Segments
Unfiltered	Continuously annotated	3^a^	3^a^	12.9	9,296
	Randomly sampled	3^b,c^	3^b,c^	3.2	2,326
Filtered	Detector selected	14^b,d^	14^b,d^	24.9	17,917
	Human selected	12^a,c,d^	12^a,c,d^	13.6	9,779
**Total**		**22**	**22**	**54.6**	**39,317**

Bold is for emphasis.

^a^ Note that 2 participants in the Continuously Annotated Unfiltered set were also included in the Human Selected Filtered set.

^b^ Note that 3 participants in the Randomly Sampled Unfiltered set were also included in the Detector Selected Filtered set. The segments annotated for these 3 participants in both sets differ but may have some overlap.

^c^ Note that 1 participant in the Randomly Sampled Unfiltered set was also included in the Human Selected Filtered set.

^d^ Note that 5 participants in the Detector Selected Filtered set were also included in the Human Selected Filtered set.

*Unfiltered set:* The Unfiltered set is created by directly annotating subsamples of daily audio recordings in two ways: 1) by continuously annotating portions of the daily recordings forming the Unfilt-Continuously Annotated set, and 2) by randomly sampling segments from the recordings and annotating those segments, forming the Unfilt-Randomly Sampled set. The complete Unfiltered set is used in the development and assessment of our High Chaos Detector, and is further detailed in Section 4.2 below.

*Filtered set:* We also employ two filtering strategies, 1) our High Chaos Detector and 2) Human Selection to more efficiently generate a substantial training and testing dataset, together comprising our Filtered set. As detailed in Section 4, the detector is used to identify candidate segments likely to contain chaotic sounds which are subsequently annotated by trained research assistants. Similarly, human selection is used to identify additional candidate *no chaos/silence* and *high chaos* segments (Section 5.2.1) which are later annotated.

We combine the Unfiltered and Filtered sets into the Annotated dataset that is used to train and test the auditory chaos multi-class classifiers, as detailed in Section 5.

## 4. High chaos detector

Our first aim was to develop a high chaos detector to aid in efficiently annotating rare high chaos (Chaos level 3) events with the goal of creating a balanced training dataset for modeling. Our detector selects candidate segments for manual annotation. Candidate segments have an increased likelihood of containing ground truth high chaos events as determined by the presence of loud, jarring or otherwise stimulating sound classes. To obtain candidate segments, we leverage an existing everyday sound classifier that can detect 521 sounds classes of various levels of stimulation (e.g. silence or white noise vs. restaurant sounds or dishes clanking) which we use to map audio segments to our four chaos levels (see Table 1). The logic of the detector is that mapping a near-exhaustive list of everyday sound predictions to chaos levels will aid in identifying high chaos candidate segments. Identifying candidate segments increases the annotation efficiency by reducing the annotation set, as only those segments predicted to contain *high chaos* are manually annotated for four levels of auditory chaos. After annotating the candidate high chaos set, we found that all four chaos levels including high chaos had sufficient variability of chaos classes annotated to form a balanced dataset to train and test our model. As such, we did not label additional data for other levels of chaos.

Below we describe the development, implementation, and evaluation of the detector. It is important to note here that the main goal of the detector is to maximize the recall of high chaos events while decreasing the size of the candidate set needing to be annotated. The precision of the detector helps decrease the size of the candidate set; given the complex nature of auditory chaos, we define the detector to be successful as long as the size of the candidate set is smaller than the original dataset and we get a reasonably good amount of labeled high chaos segments.

### 4.1. Development and implementation

**Figure 2:**
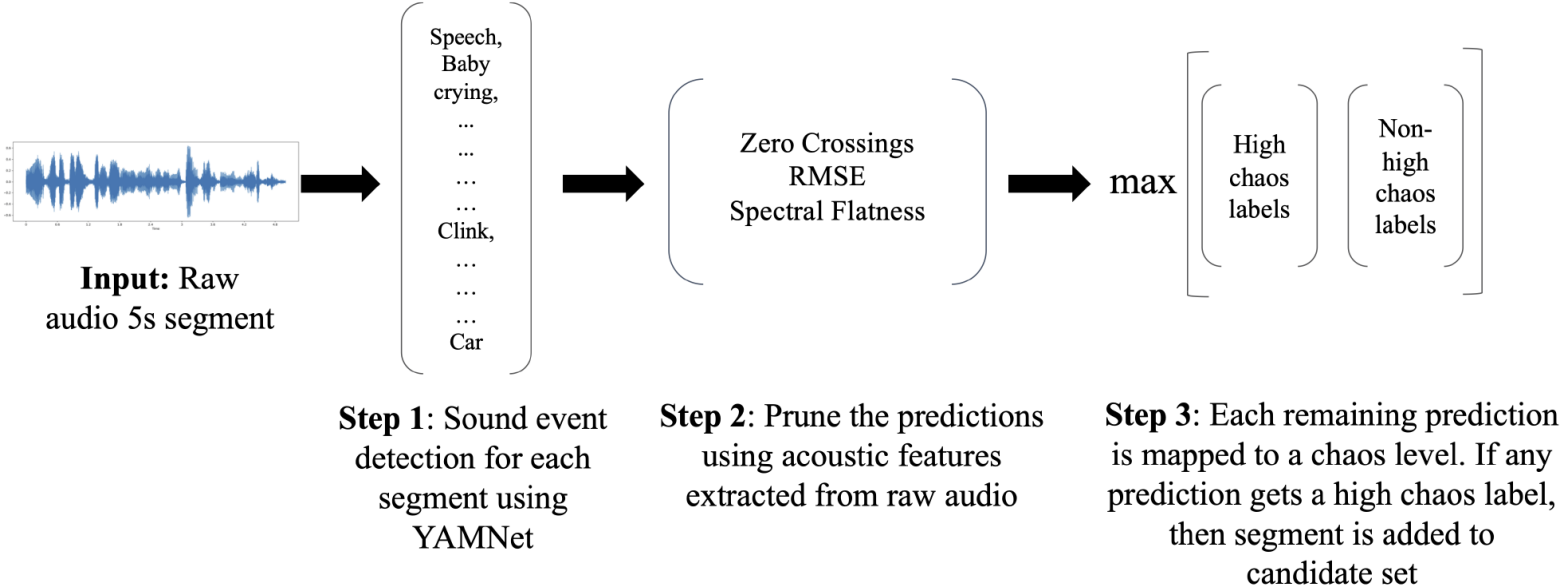
Step-by-step workings of the high chaos detector starting from a raw audio input segment to the predicted chaos classes.

The high chaos detector leverages a publicly-available audio classifier, YAMNet (Yet Another Mobile Network) by Google (41), to sample candidate audio segments for high chaos. Figure 2 illustrates the pipeline for our high chaos detector, which we detail in the text below.

#### 4.1.1. Clustering YAMNet classes into high chaos and non-high chaos groups

YAMNet is a pretrained classifier employing the MobileNetV1 depthwise-separable convolution architecture (73). It is trained on Audio Set (48) and can classify 521 everyday audio events. YAMNet takes as input raw audio segments of any fixed length (minimum 975 ms). Audio segments are resampled to 16 kHz mono and then converted to mel-spectrograms before being passed to the model. YAMNet then outputs 521 per-class output scores of the predicted sound events for the entire input audio segment.

To leverage YAMNet predictions to automatically sample candidate high chaos segments, we first manually grouped the individual YAMNet classes into two groups—highly chaotic and not highly chaotic sounds. To reduce the rate of erroneous predictions, out of the 521 YAMNet classes, we did not consider those that had a quality estimate of 33% or below in Audio Set. Quality estimates are provided by Audio Set as a measure of the accuracy of their annotated labels. Furthermore, we also excluded classes that we determined would be unlikely to be present in our infant-worn daylong recordings (e.g. eruption, artillery fire, motorboat/speedboat), leaving us with 368 YAMNet classes. Next, we manually grouped each of these 368 classes into high chaos and non-high chaos groups. These labels were determined by common associations with the sound class, e.g *children shouting, shatter, bark, etc.* were labelled as *high chaos* whereas *white noise, shuffling cards* were labelled as *non-high chaos*. Additional examples of Audio Set classes predicted by YAMNet assigned to the different levels of chaos can be found in Table 1. Chaos 0, 1, and 2 fall in the non-high chaos group and Chaos 3 represents the high chaos group.

#### 4.1.2. Pruning YAMNet predictions

During qualitative assessment of the accuracy of YAMNet predictions on our infant-worn audio data, we identified two classes that were frequently incorrectly predicted by YAMNet. First, YAMNet frequently misidentified positive or neutral infant vocalizations or babbling as *infant crying* and vice-versa. Thus, for any segment where YAMNet predicted both infant crying and babbling, we applied a heuristic to determine which was more likely. Specifically, given that crying typically has much higher root-mean-square-energy (RMSE) values than non-cry vocalizations, if *any* of the extracted RMSE values for that segment were more than 3 times the mean RMSE for that participant, we kept infant crying and dropped infant babbling and vice-versa. RMSE values were extracted for each segment using a sliding window approach with a window length of 512 samples with a hop length of 256 samples at the sampling rate of 22,050 Hz using the Librosa (74) library in Python, giving us a total of 431 RMSE values for our 5 s audio segment. Mean RMSE for a participant was calculated by taking the average of all extracted RMSE values (using the above sliding window approach) for all segments in the entire daylong recording from that participant.

YAMNet was also unable to distinguish between *vehicles* and background *white noise* sounds commonly used to facilitate infant sleep. As white noise sounds are typically quieter and have a flatter waveform than vehicles, we used spectral flatness and zero crossings to distinguish them. Similar to RMSE, we extracted spectral flatness values for each segment using the sliding window approach, giving us 431 spectral flatness values for each 5 s segment. Zero crossings were computed at the segment level as the total number of times the audio signal crossed from positive to zero to negative or negative to zero to positive during the five second duration of the segment. If any segment had *all* spectral flatness values greater than 0.0001 or the number of zero crossings were between 1000 and 4000 (corresponding to unvoiced noisy audio) and the segment had a predicted label *vehicles* or similar, we dropped it.

#### 4.1.3. Leveraging YAMNet predictions for automatic high chaos detection

To automatically sample candidate segments for high chaos, we first provided our raw 5 s audio segments to YAMNet to obtain sound event predictions. YAMNet provides a confidence level for each of its predictions. We only considered the top ten predictions based on the confidence level and discarded all predictions below 0.01% confidence. Next, we additionally pruned these predictions using acoustic features as described above. Finally, for each segment, all remaining YAMNet predictions were assigned a high chaos or non-high chaos group according to the groups created above. If any of the YAMNet predictions for a segment were mapped to a high chaos group, the segment was chosen as a candidate segment for high chaos by the detector. Furthermore, to circumvent YAMNet’s missed or erroneous predictions, and to ensure that we captured all high chaos segments, we included all segments in the high chaos candidate set irrespective of their YAMNet predictions if any of their extracted RMSE values using the sliding window approach were more than 7 times the mean RMSE for that participant i.e. very loud segments. Qualitative trial-and-error analyses were used to determine the threshold for identifying very loud segments.

### 4.2. Dataset

We evaluate the performance and efficiency of the detector in identifying high chaos events using both subsets of our unfiltered annotated data: continuously annotated data and randomly sampled data (see also, Figure 1, top pathway).

#### 4.2.1. Unfiltered set: Continuously Annotated data (Unfilt-CA)

To test the performance of our detector for identifying high chaos events, we annotated continuous 2.6 to 7 h segments from three unique participants’ daylong recordings, totaling 12.9 h of annotated data (9296 5 s segments). This Continuously Annotated data is a good representation of the chaos present in continuous daylong audio recordings. We also use this dataset to assess the feasibility of obtaining a sufficient sample of rare high chaos events using typical annotation strategies.

#### 4.2.2. Unfiltered set: Randomly Sampled data (Unfilt-RS)

To test the efficiency of the detector for identifying high chaos events, we compared the proportion of ground-truth high chaos annotated in high chaos candidates (identified by the detector) with randomly sampled segments from the same participants. In a sample of 3 participants, we matched the number of randomly selected segments to the number of candidate high chaos segments labeled by the detector for that same participant. For example, if for one participant, the detector identified 100 segments as high chaos, we randomly sampled 100 segments of raw audio data as a comparison from the same participant. In total, 3.2 h of data (2326 5 s segments) were randomly sampled from 3 participants and annotated by the trained research assistants for four levels of chaos. These annotated segments form the Randomly Sampled dataset.

### 4.3. Evaluation

Our detector had a recall of 0.653 and a precision of 0.267 for the high chaos class (Chaos 3), as evaluated on the Continuously Annotated data. This means that we missed 34.7% of high chaos events present in the raw data. However, given that the goal of our detector was to increase annotation efficiency of these relatively rare high chaos segments, we find our detector’s performance adequate. Specifically, the detector allowed us to annotate only 9.85% (24.8 h) of the entire daylong recordings from 14 participants (244.3 h) while providing about 4 h of ground truth *high chaos* positive examples.

Next, we evaluated the extent to which the detector increases annotation efficiency of the rare high chaos events. To do so we compared the proportion of ground-truth high chaos segments identified in randomly sampled data vs. segments identified as high chaos by our detector. 16.8% of detector-identified high chaos segments were labeled as *high chaos* in ground truth annotation, vs. only 2.02% of the set sampled by random sampling. Thus, the detector identified 8.32 times more *high chaos* events in a matched volume of audio randomly drawn from the same three participants’ recordings.

## 5. Auditory chaos classifiers

Distinguishing between levels of auditory chaos depends upon many factors including the volume, quantity, and quality of sounds, the source and type of sounds, and the extent of overlapping sounds. We explored multiple different machine learning models to solve this task. Given the complexity of chaos classification, a deep learning approach where the model identifies and learns the most distinguishing features, may perform better than a traditional machine learning model that requires human feature engineering. When applied to a variety of audio recognition tasks, deep learning models have repeatedly shown superior performance in comparison to traditional models (60, 22, 75). However, there is no prior work in the domain of auditory chaos classification. Therefore, we evaluate and compare the performance of a traditional machine learning model, namely Random Forest (RF), trained using a range of classical acoustic features, and a deep learning framework, Convolutional Neural Network (CNN). Additionally, given that volume has been used as a proxy for household chaos (36), to provide additional justification for our work, we train a baseline model, a RF, using audio volume features only.

Our goal is to train a model to classify a given input audio segment into four levels of auditory chaos. To train our classifiers, we used both filtered and unfiltered annotated data (i.e. the *Annotated dataset*). As is standard, we tested our models on the Annotated dataset as well in a leave-one-participant-out cross-validation (LOPO-CV) fashion. Additionally, we tested our models on subsets of our unfiltered ground truth data to evaluate if model performance generalizes to real-world scenarios and daylong audio recordings. Finally, we explored if human annotation time and effort can be minimized by investigating the relationship between size of training data with model performance.

### 5.1. Model development and implementation

#### 5.1.1. Baseline model with volume features only: RF-3f

We developed a baseline model to test whether volume features alone could be used to predict four ground truth levels of auditory chaos. For each 5 s audio segment that was annotated for ground truth auditory chaos (detailed in Section 5.2), we extracted the peak amplitude and RMSE features, to represent the loudness or energy of that audio segment. We evaluated if peak amplitude and RMSE had the predictive power to successfully classify ground truth chaos levels using a RF. For each audio segment, RMSE was extracted using a sliding window approach for a window size of 512 samples with a hop length of 256 samples and the mean and standard deviation across the 5 s segment was computed and used as features. Peak amplitude was computed by taking the maximum amplitude in the 5 s audio segment. These three features were fed as inputs to the RF (model referred to as RF-3f) with 1000 estimators and the model performance was assessed. All features were extracted using the librosa (74) library in Python.

#### 5.1.2. Traditional acoustic features model: RF-53f

In the traditional machine learning approach, we extracted a broad range of classical acoustic features from the raw audio segments and fed them as inputs to the RF. For each 5 s audio segment, we extracted 27 features comprised of 20 MFCCs, zero crossing rate, spectral features (flatness, rolloff, centroid, bandwidth), RMSE and peak amplitude using librosa. These features were chosen as they have been successfully used in previous works for sound event detection (22, 58, 54, 52) and scene classification (51, 76, 77), domains most similar to auditory chaos classification. Similar to our baseline models, all features were extracted using a sliding window approach for a window size of 512 samples with 50% overlap. Mean and standard deviation for 26 out of the 27 features (except peak amplitude) were computed across the 5 s segment, giving us a total of 53 (26∗2+1) features. These 53 features were fed as inputs to the RF (model referred to as RF-53f) with 1000 estimators and the model performances were assessed.

#### 5.1.3. Deep learning model: CNN

Our deep learning model is taken from a previously published work in the sound event classification literature (42). We chose this model because the previous work has showcased that it has good performance when trained from scratch for multi-class sound event classification—a domain most related to auditory chaos classification. Moreover, the training dataset used to train the model in (42) consists of 41.2 h of audio data, very similar to our 40 h of balanced chaos training data. This ensures that the model complexity (in terms of number of convolutional layers) is appropriate for the amount of chaos training data we have and the model will not overfit or underfit our training data. We train and test this network with our real-world first-person infant-centric auditory chaos data.

The model employs a Convolutional Neural Network (CNN) with three convolutional layers (5×5 kernel), incorporating Rectified Linear Unit (ReLU) activations. Two max-pooling operations are interleaved with these convolutional layers. Additionally, Batch Normalization (BN) layers are placed before each convolutional layer, followed by ReLU activation. At the network’s terminus, two fully connected (dense) layers are added. To further enhance model performance, the established pre-activation technique is implemented, where BN and ReLU activation are applied before each convolution operation. Figure S1 in the Supplementary Materials depicts the model architecture along with it parameters. The model has ≈0.5M weights. It uses the categorical cross-entropy (CCE) loss function, a batch size of 64 and an Adam optimizer with an initial learning rate of 0.001 along with Earlystopping applied with a patience of 15 epoch based on the validation accuracy. All hyperparameters mentioned above were kept exactly the same for our chaos model except the input audio segment length was changed to 5 s to match the length of our audio segments.

The 5 s raw audio segments are chunked into 2 s patches and these patches are converted to log-scaled mel-spectrograms with 96 components (bands) using a window size of 40 ms with 50% overlap, to be fed as input to the model. Patches which are shorter than 2 s are replicated until the desired length of 2 s is reached. Each patch retains the segment level ground truth chaos label. The chaos model outputs segment-level predictions from our four chaos classes (0–3) which are obtained by computing predictions at the 2s patch-level and aggregating them using geometric mean.

### 5.2. Dataset

To train and test our classifiers, we combined our filtered and unfiltered sets to create our Annotated dataset. In total, the Annotated dataset comprised approximately 55 h of labelled data across daylong recordings of 22 participants. Table 3 provides a summary of the subsets of data that comprise the complete Annotated dataset. For model training, we subsampled a balanced set from the Annotated dataset, as detailed below.

#### 5.2.1. Filtered set

The Filtered set combines two filtered sets, the Detector Selected set (DS-Filt) and the Human Selected set (HS-Filt), (see also Figure 1, pathways two and three). Together, the Filtered set comprises a total of 38.5 h (27,696 5 s segments).

DS-Filt was created by manually annotating all candidate high chaos segments identified by the detector in the daylong recordings of 14 participants, including the 3 participants in the Randomly Sampled set. The candidate set containing 17917 segments (24.9 h) was annotated by trained research assistants for four levels of chaos. While filtration successfully increased the proportion of high chaos in the training dataset, overall the filtered data was heavily biased towards *low* and *medium* chaos segments, which made up 85% (20.4 h) of the annotated segments. By contrast, *high* and *no* chaos segments comprised approximately 12% (4.4 h) and 3% (0.14 h) of the filtered dataset, respectively.

HS-Filt was created to further increase the amount of training data, specifically for the *high* and *no* chaos classes. Human annotators identified and annotated an additional set of audio segments from recordings containing high levels of *no* chaos and *high* chaos. We achieved this through various means, including by selecting recordings where families shared with us that they recorded at special events or locations that may be particularly chaotic, including museums, restaurants, or daycare settings, as well as by listening to parts of the recording to attempt to identify extended periods of time (0.2–2.7 h) that contained these classes of chaos. In this manner, we annotated 8.3 h of *no* chaos and 5.2 h of *high* chaos. This gave us a total of 13.6 h (9779 5 s segments) of annotated data from 12 participants (including 3 participants from the Unfiltered set and 5 participants from DS-Filt).

#### 5.2.2. Unfiltered set

The Unfiltered set combines the Continuously Annotated (Unfilt-CA) and Randomly Sampled (Unfilt-RS) sets used to evaluate the high chaos detector, as detailed in Section 4.2 above (see also Figure 1, top pathway). In total, the Unfiltered set comprised of 16.1 h (11622 5 s segments) of annotated data.

#### 5.2.3. Creating a balanced dataset for model training

As our Annotated dataset was imbalanced, prior to any and all model training we subsampled this dataset to create a balanced training dataset. Specifically, the complete Annotated dataset included: 8.7 h of *no chaos*, 16.3 h of *low chaos*, 19.0 h of *medium chaos*, 10.7 h of *high chaos* totalling 54.6 h. Thus, the maximum amount of balanced data we could use to train our auditory chaos model was 40 h (10 h per chaos level), limited by the amount of high chaos annotated minus the test set. We did not want to sample with repetition for any of the chaos levels other than *no chaos*. *No chaos* denotes complete silence or absence of any sounds, so sampling with repetition is less likely to change the nature of the class. To ensure that every *no chaos* segment annotated was included in the training set atleast once, all the annotated *no chaos* data from the non-test participants was included in the train set and the amount of *no chaos* data needed to make it 10 h was sampled with repetition. For chaos levels, where more than needed data was available, the required hours were randomly sampled.

### 5.3. Evaluation

We conducted five analyses to evaluate model performance under different conditions. For each analysis, the models were tested using LOPO-CV. For each fold of LOPO-CV, models were trained with a set of data balanced across all four levels of chaos, randomly sampled from the Annotated dataset (minus the test participant’s data) as described in Section 5.2 and tested on the test participant’s data. The participants as well as their data included in the test set varied depending on the analysis as detailed below.

As our datasets are highly imbalanced, we report both macro and weighted evaluation metrics. We calculated both *global macro* and *global weighted* F1 scores to assess the model’s performance across the entire test set. Global performance metrics take into account all instances in the test set, providing a single aggregated metric for the entire dataset. Additionally, we computed *participant-specific weighted* performance scores in order to statistically test the differences between pairs of models on individual participants using paired t-tests. Given heavily skewed chaos class distributions and small participant-specific datasets, individual participants often had very few samples for some chaos classes e.g. less than 60 5 s samples i.e less than 5 min of data. This led to highly noisy, non-representative performance on these minority chaos classes, biasing the overall evaluation metrics. As a result, we refrained from computing *participant-specific macro accuracy scores* to ensure a more accurate representation of our model’s real-world performance.

**Figure 3:**
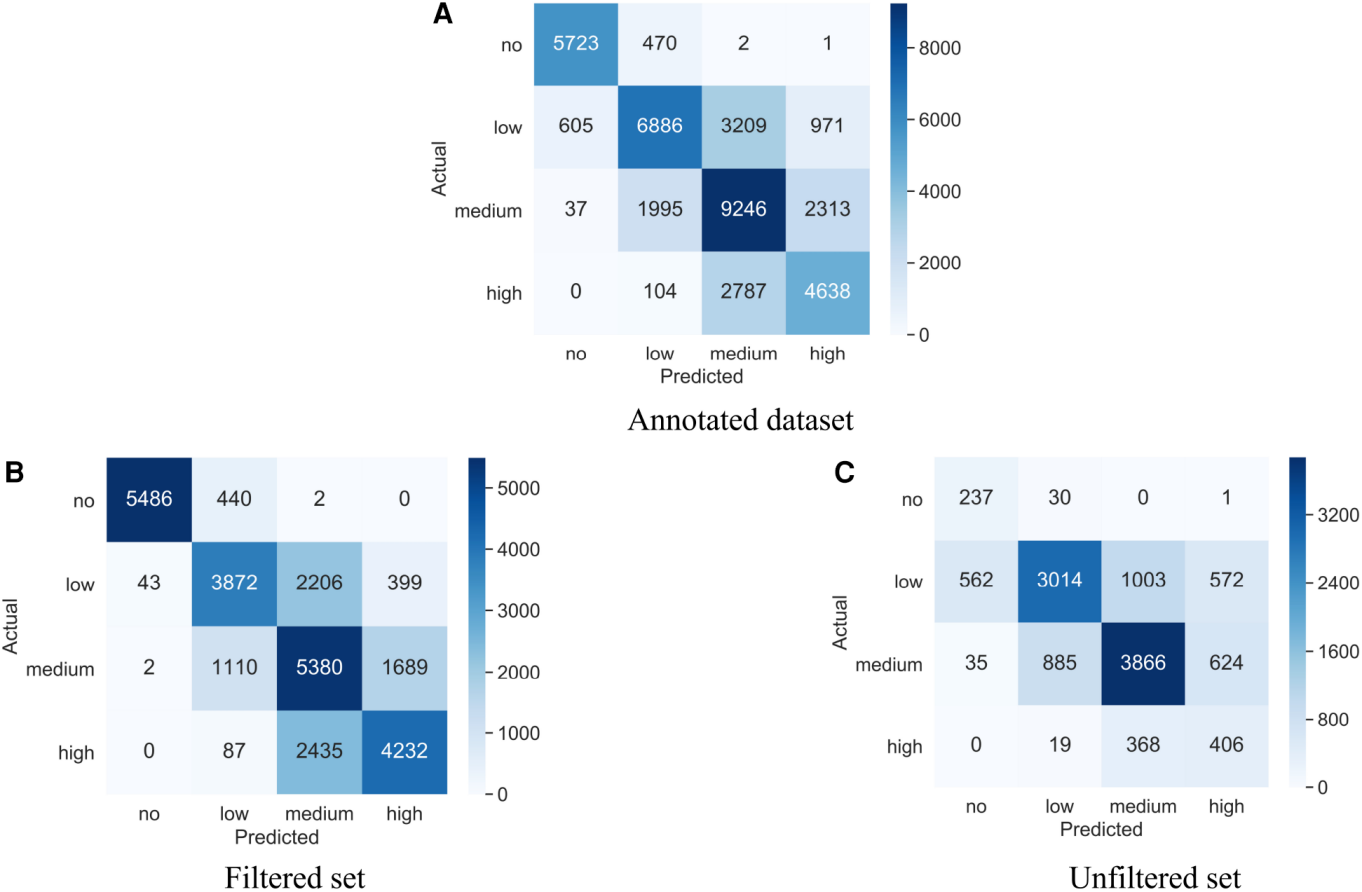
Confusion matrices for our best-performing auditory chaos CNN model (**A**) Trained and tested on the Annotated dataset across 22 participants. (**B**) Trained on the Annotated dataset; tested on the Filtered set of 21 participants. (**C**) Trained on the Annotated dataset; tested on the Unfiltered set of 6 participants.

Table 4 summarizes the global macro and weighted performance of all three models on the different test sets. Figure 3 provides the confusion matrices for our best-performing CNN model on the Annotated dataset as well as its two component sets, the Filtered set and the Unfiltered set. Participant-specific weighted performance metrics, along with complete results for paired t-tests are summarized in Table S1 and Section 2 of Supplementary Materials, respectively.

**Table 4:** Global macro and weighted model performance for our three models on different test sets.

Models	Test data	Macro			Weighted		
		F1	Precision	Recall	F1	Precision	Recall
RF-3f	Annotated (Unfilt. + Filt.)	0.267	0.269	0.266	0.284	0.291	0.278
	Filtered (DS + HS)	0.265	0.267	0.267	0.269	0.269	0.271
	Unfiltered (CA + RS)	0.240	0.279	0.333	0.338	0.448	0.295
	Cry Set	0.352	0.357	0.368	0.411	0.451	0.394
	Non-cry Set	0.249	0.253	0.247	0.264	0.271	0.259
RF-53f	Annotated (Unfilt. + Filt.)	0.616	0.676	0.592	0.611	0.639	0.614
	Filtered (DS + HS)	0.597	0.660	0.582	0.589	0.641	0.586
	Unfiltered (CA + RS)	**0.560**	0.562	0.592	**0.676**	0.678	0.682
	Cry Set	0.626	0.655	0.608	0.666	0.669	0.669
	Non-cry Set	0.594	0.673	0.575	0.599	0.646	0.601
CNN	Annotated (Unfilt. + Filt.)	**0.701**	0.705	0.702	**0.679**	0.685	0.680
	Filtered (DS + HS)	**0.710**	0.725	0.701	**0.697**	0.708	0.693
	Unfiltered (CA + RS)	0.539	0.510	0.674	0.665	0.706	0.647
	Cry Set	**0.646**	0.657	0.644	**0.680**	0.687	0.681
	Non-cry Set	**0.692**	0.696	0.694	**0.681**	0.690	0.680

*Note:* Models were trained using 40 h of balanced data across four levels of auditory chaos randomly sampled from the Annotated dataset and evaluated using LOPO-CV on their respective test sets. Global macro and global weighted F1 score, precision and recall were computed using the chaos predictions and ground truth chaos labels for the entire test set. Results for each analysis are separated using emphasis lines. Model performance in bold represents the highest F1 score achieved across all three models for that particular test set.

#### 5.3.1. Model performance on Annotated dataset

On the Annotated dataset, the CNN model achieved the highest F1 score across all three metrics, followed by the Acoustic features RF-53f model. However, when the participant-specific weighted F1 scores were compared using paired t-tests, these two models were not statistically differentiated from one another. The baseline model, RF-3f, had substantially worse performance than the CNN and the RF-53f models across all three metrics and the difference was significant in terms of participant-specific weighted F1 scores.

#### 5.3.2. Model performance on Filtered set

The pattern of results on the Filtered set mirrored those of the Annotated dataset, with the CNN having higher performance than the Acoustic features RF-53f model in terms of all three metrics, with insignificant differences between CNN and RF-53f considering participant-specific weighted F1 scores. Both these models exhibited superior performance across all three metrics compared to the baseline model, RF-3f, showing a significant variance in participant-specific weighted F1 scores.

#### 5.3.3. Model performance on Unfiltered set

To ensure that our models generalize to daylong recordings, i.e. our domain of interest, we tested the above model performances on unfiltered data, i.e. that was not sampled by the detector or human sampling. This provides a truer representation of the chaos present in daylong recordings. In contrast to the prior results, the Acoustic features RF-53f model had higher accuracy than the CNN model on global macro and weighted metrics. However, the CNN had the highest participant-specific weighted F1 score. Again, the CNN model was not statistically differentiable from the RF-53f using the participant-specific weighted F1 scores. As above, both the RF-53f and the CNN substantially outperformed the baseline model, RF-3f in terms of all three metrics and their performance was significantly higher with regards to the participant-specific weighted F1 score.

#### 5.3.4. Model performance on Cry and Non-cry sets

As infant crying is likely to occur in our infant-worn audio recordings and could contribute a substantial proportion of high chaos labels, we tested the performance of our models on both cry and non-cry audio segments. Knowledge of our model performance on the non-cry segments is important for research questions examining impacts of chaos on infant crying and vice versa, as well as more broadly for researchers who want to distinguish chaotic sounds that originated from the target child vs. those that originated elsewhere. We used the YAMNet *infant crying* class to identify all segments that included infant crying in the Annotated dataset. Cry labels were used to split the Annotated dataset into two subsets—Cry and Non-cry set. To ensure accurate evaluation, in the Cry set, we dropped segments predicted as Chaos 0. The Cry set included no ground truth Chaos 0 segments and less than nine predicted Chaos 0 segments, meaning we did not have sufficient segments to assess performance in this class which would otherwise bias our global macro metrics. Global evaluation measures were then computed across all 22 participants for the Cry and the Non-cry sets separately. The CNN model again performed better than the Acoustic features RF-53f model on both the Cry and Non-cry sets. Both models performed substantially better than the baseline model, RF-3f. Confusion matrices for the CNN model can be found in Supplementary Figure S2.

#### 5.3.5. Effects of training data ablation on best-model performance

**Figure 4:**
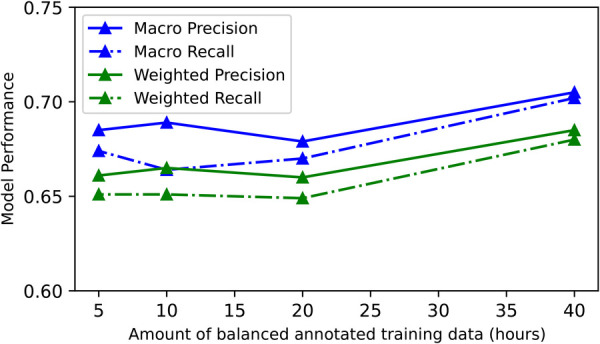
Results of the training data ablation study for our best-performing CNN model—global model performance on the Annotated dataset as a function of training data (Unfiltered + Filtered set). The model performance drops as we decrease training data.

To examine model performance as a function of the size of training data we conducted a data ablation study. As the CNN model had the highest F1 scores on 4 out of 5 test sets including the Annotated dataset, our largest test set, we used this model to conduct our data ablation study. We ran 12 experiments (3 runs × 4 training data sizes) varying the amount of training data sampled from the Annotated dataset. We used a range of exponentially decreasing balanced sets, specifically: 40, 20, 10 and 5 h. For all experiments, we trained and tested the CNN using LOPO-CV across all 22 participants. When trained with 5h of balanced data, the model achieved a global macro precision of 0.685, recall of 0.674 and F1 score of 0.674 and a global weighted precision of 0.661, recall of 0.651 and F1 score of 0.649. Adding 35 additional hours of annotated training data (40 h total) improved the macro precision by 0.020, recall by 0.028 and F1 score by 0.027. Similarly, the global weighted precision, precision and F1 score were improved by 0.024, 0.029 and 0.030 respectively. Therefore, both global macro and weighted metrics improved after the addition of more training data. Figure 4 showcases the effect of training data ablation on the CNN model performance (exact model performance values can be found in Table S2 in Supplementary Material).

## 6. Discussion

To facilitate research and intervention on the effects of household chaos on child functioning (2–4, 7–11), we developed and compared various multi-class classifiers for detecting auditory chaos in real-world settings. To efficiently annotate rare high chaos events, we developed a high chaos detector, which resulted in an 8.32× increase in efficiency in identifying these events relative to baseline rates. Our best-performing auditory chaos model—a CNN trained with 40 h of balanced annotated real-world data– achieved a macro F1 score of 0.701 and a weighted F1 score of 0.679 in challenging real-world settings.

### 6.1. CNN achieves best overall model performance

We tested three different models for auditory chaos classification. Our results indicate that the deep learning approach using a CNN architecture achieved the highest performance in terms of global macro and weighted F1 score in 4 out of 5 test sets. The acoustic features model trained on 53 features (RF-53f) had the highest performance in terms of global metrics on the remaining test set, the Unfiltered set (see Discussion in Section 6.2, below). However, when participant-specific weighted performance metrics were computed for all models, CNN had the highest performance across all test sets. We note that while performance values differed across the CNN and acoustic features model, paired t-tests comparing participant-specific weighted F1 scores, indicate that these differences were not statistically *significant* (see Supplementary Section 2). That is, the CNN and RF-53f appear to be statistically equivalent models for classifying auditory chaos. However, as the CNN model achieved the highest performance on the Annotated dataset, our largest test set, and the majority of the test sets, we recommend that future users interested in automated auditory chaos detection use our CNN model. We, therefore, make the trained CNN model publicly available on Github for future applications.

**Figure 5:**
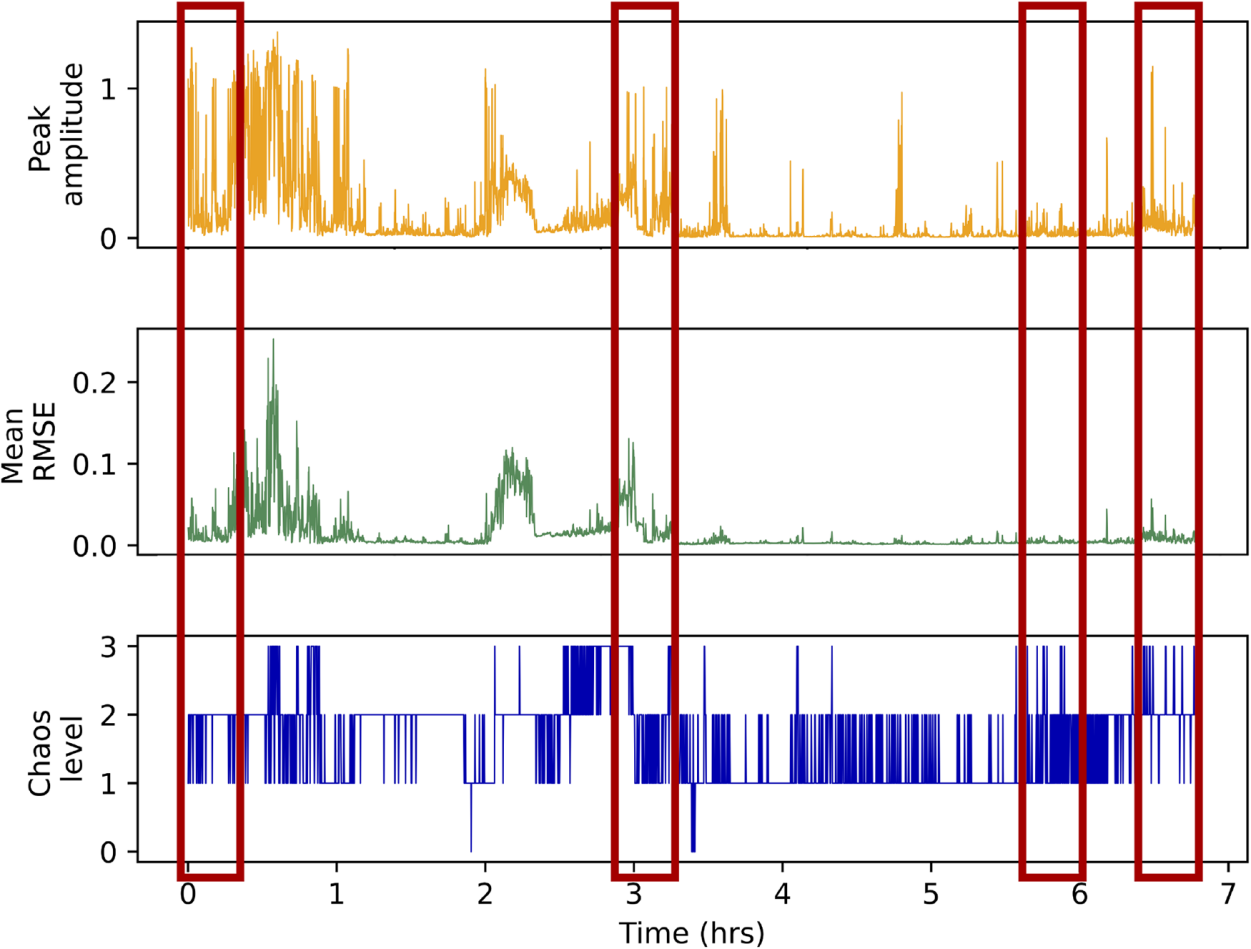
Peak amplitude (orange spikes), mean RMSE (green spikes) and ground truth auditory chaos levels (blue spikes) for each 5 s audio segment annotated from one participant’s audio recording. The x-axis represents the audio segment number. Each hour has 720 5 s audio segments so the data represented here is ∼7 h of continuous audio recording. The red boxed regions highlight sample regions of the recording where volume is high, as indicated by high peak amplitude and/or mean RMSE values, but a segment is not annotated as high chaos (level 3) or vice-versa. This illustrates that features representing audio volume are not consistently able to distinguish between the four levels of chaos, which we we also documented using our baseline models.

Unsurprisingly, the baseline model trained with three volume-related features had substantially and significantly lower performance than both the CNN and the more comprehensive acoustic features model, RF-53f (see Supplementary Section 2 and Table S1 for t-test results). Overall, it appears that volume alone cannot be used to distinguish between the four different levels of chaos. Figure 5 additionally illustrates this point by visualizing volume features and annotated chaos labels from 7 h of continuous audio recording shared by one of our participants. These results indicate the value of developing a model for the auditory chaos classification task rather than relying on markers of audio volume for characterizing auditory household chaos.

### 6.2. Model generalization from filtered to unfiltered data

Training a model with filtered data can reduce the model’s performance on raw or unfiltered recordings which provide a “truer” or less biased representation of chaos found in real-world everyday recordings. However, assessing our model’s performance on unfiltered real-world data is challenging due to the lack of a large-enough, representative ground truth dataset, especially for the rarer chaos classes. Thus, while we evaluate the generalizability of our model on our unfiltered dataset, we remind our readers that this dataset comprises 16.1 h of data annotated from 6 participants and includes approximately 1 h of *high chaos* data and less than an hour of *no chaos* data. Thus, it is unlikely to capture the full distribution of chaos in everyday settings, as we elaborate below.

All models showed worse global macro F1 performance on our Unfiltered set relative to the Filtered set and the complete training dataset including filtered and unfiltered data (Annotated dataset). This suggests that our models, whose training data included ∼70% filtered data, may not fully generalize to unfiltered data. Our acoustic features model (RF-53f) showed relatively similar global macro F1 performance on filtered and unfiltered data, within .04 points of one another. However, our CNN model showed a 17.1 point drop in global macro F1 score between filtered and unfiltered data. This may reflect that the CNN model overfit more to the training data compared to RF-53f. Due to its higher model complexity, the CNN model may have overlearned the characteristics of sound events contained in the filtered segments with reduced generalizability to the Unfiltered set. By contrast, the relatively less powerful RF may have less capacity to learn more complex features to distinguish between the chaos classes and thereby generalized better to the Unfiltered set.

Next, as illustrated by the confusion matrix in Figure 3C, our CNN model had relatively low performance for the minority Chaos 0 (no chaos) and Chaos 3 (high chaos) classes in the Unfiltered set in particular. This trend is also apparent in both RF models. The models’ relatively low performance on *high chaos* and *no chaos* classes in the Unfiltered set could be due to the fact that these classes were by far the rarest classes in the unfiltered dataset. As such, their ground truth training data was more likely to be obtained through the use of filters, relative to more common Chaos 1 and 2 classes. Incorporating filtered ground truth data allowed us to efficiently provide the model with large volume and variety of ground truth training data. However, these filtered data may have some biases. For example, the *high chaos* samples selected by the detector might not encompass all high chaos sound events occurring in infant’s everyday environments. Additionally, filtered high chaos segments selected by human sampling may have been easier for the model to classify given that they lasted many minutes (e.g. ambient sounds from a party or daycare center), versus only a few seconds, e.g. a yell, a loud bang, a bark, etc. Thus, one possibility is that the Unfiltered set contained more “difficult-to-identify” high chaos events relative to our Filtered set, contributing to challenges with generalizibility. However, as noted above, the limited size of the Unfiltered set raises the concern that it does not provide an accurate representation of real-world chaos. As such, while potentially biased, our much larger Annotated dataset (54.6 h; 22 participants), which included over 7680 5-second high chaos segments, is likely to be providing more robust performance measures than our Unfiltered set, in particular for rare Chaos 0 and Chaos 3 classes.

### 6.3. Model performance is consistent with other real-world models

All model results were achieved in completely unstructured, real-world audio data from recordings worn by infants in their everyday home environments. As this is the first published work for developing an auditory chaos classifier, our work represents a baseline model for future efforts. Relatedly, there is no benchmark to compare our models with directly. We note that published audio detection models trained on clean, lab-collected dataset or synthetic datasets often achieve accuracies in the 0.90 s. However, it is well established that models trained on such “clean” datasets do not generalize to noisy real-world scenarios (22, 38, 37, 59). By contrast models trained and tested on real-world audio data generally show substantially lower accuracies, with F1 scores often around 0.6–0.7 (37, 38, 78). For example, a recent analysis of LENA, a widely used platform for speech detection and speaker classification from child-worn audio recordings, reported precision and recall values ranging from 0.27 to 0.60. Similarly, a real-world cry detection model recently developed by Yao et al. (22) achieved an F1 score of 0.613. Thus, while not directly comparable to these models, our CNN model performance falls in the range of recently published real-world sound event detection models.

Additionally, we note that real-world models with accuracies in the range of 0.60 have made real contributions to empirical research in child development. For example, measures of overheard speech derived from LENA’s speech classifier which has an overall weighted accuracy of 67% (79) have predicted various measures of language development in young children. A review paper provides a summary of works that used LENA’s in-built algorithms to detect aspects of the speech environment and were found to significantly predict individual differences in child language development as well as gold-standard laboratory measures (80). These examples indicate that model results much lower than those obtained in clean laboratory conditions can be of value to the developmental psychology community.

### 6.4. High chaos detector increases annotation efficiency

Our high chaos detector was able to identify 65.3% (recall) of the ground truth high chaos segments in the unfiltered Continuously Annotated set. Given that our goal was to maximize the amount and variety of high chaos ultimately annotated, a high recall value is optimal. Still, the detector missed 34.7% of the ground truth high chaos in the Continuously Annotated set. This could be due to the strategy implemented by the detector. The detector leverages a publicly-available everyday sound classifier, YAMNet. The detector’s ability to identify high chaos events is largely dependent and limited to the variety and number of highly chaotic sound classes that YAMNet can detect. Moreover, YAMNet’s performance on each of the classes it can detect also largely drives the high chaos detector’s accuracy. High chaos sound events outside of the range of YAMNet’s output classes could also contribute to the missed 34.7% of high chaos segments.

Next, the precision of our detector for the high chaos class was relatively low (26.7% on Continuously Annotated set), meaning that the detector over-identified candidate high chaos segments. This precision is comparatively lower than precision of 36%–49% reported in Audio Set’s original paper (48), the only prior paper we are aware of that reports performance of their selected candidates sets for audio annotation. This indicates that the detector’s strategy of mapping a near-exhaustive list of everyday sound classes from YAMNet to identify high chaos events was not very precise, potentially owing to the fact that many individual sound classes may be labelled as more or less chaotic depending on their context. The detector’s low precision leads to increased annotation time, counter to our goals. However, given that occurrences of high chaos are highly rare, annotating the candidate set identified by the detector provided a huge advantage over annotating randomly sampled data. In particular, the detector allowed us to annotate 8.32 times more ground truth high chaos data than in a matched volume of audio randomly drawn from the same three participants’ recordings. Overall, given that the detector provided substantial reduction in annotation time and efforts, our detector’s performance is adequate for our goal of reducing manual annotation time and effort for the rare high chaos events.

While the high chaos detector increased the efficiency of annotating high chaos segments, we also used “human filtration” to supplement our chaos annotations. We note however that our human filtration strategy does not supplant the high chaos detector. First, we implemented this strategy mainly with participants who shared with us that they had engaged in activities or events that were particularly chaotic, meaning we had additional information on these relative to other recordings. Next, the sampled listening strategy implemented by our research team identified only chaotic activities that were at least 10 min in duration. As such, sampled listening is likely to miss shorter chaotic events, e.g. a bark, plates crashing, a scream or shout, etc and could lead to bias in the data. By contrast, the high chaos detector was able to successfully identify high chaos instances present in daily recordings irrespective of their duration.

### 6.5. Model performance across contexts and populations

Infant crying is a high chaos event likely to occur frequently in infant-worn daylong recordings and therefore our training data. As such, model performance could rely on inadvertently training a “cry detector” rather than a chaos classifier per se. To test this, we compared CNN model performance on datasets that did and did not include infant crying. Model performance was similar between Cry and Non-cry samples. Thus, our model successfully classifies the chaos level of non-cry events.

Next, in attempts to understand the shortcomings of our model, we sampled segments that the model erroneously classified. We found that our model consistently misclassified relatively loud sleep machine/white noise segments as medium chaos rather than low chaos. This was likely a result of their loud volume as sleep machines are typically kept close to the child while sleeping. Moreover, some white noise machine sounds are also acoustically very similar to high-frequency engine or mechanical tool sounds and the model was not able to differentiate between them and incorrectly identified them as medium or high chaos. As sleep machine sounds/white noise can comprise up to 12 h of an everyday recording collected via infant-worn audio sensors, this has the potential to impact the model performance significantly. Thus, we caution researchers using our model outputs on audio collected during infant sleep, in particular if families use sleep machines/white noise machines. Alternatively, researchers can ask families directly to report if they do use sleep machines.

Finally, we note that the data used to train and test this model was collected mostly from 0- to 6.5-month old infants from English-speaking families living in a mid-sized urban city and ∼60% of our participants where non-Hispanic White. Models are most likely to generalize well to populations similar to those included in the training data (81–83). Therefore, we recommend additional tests and validation before applying this model to daily recordings collected from families differing in family structure and dynamics, sociodemographic characteristics, and language from the dataset used in this study.

### 6.6. Increasing training data boosts model performance

Increasing the training data from 5 to 40 h provides a meaningful boost to the CNN model performance. Large volumes of training data are known to improve model performance (84). This may be particularly true for models designed to perform in real-world contexts with high levels of variability in class representations. We note also that the scale of the observed effect may be muted by the fact that training data for all data ablation models was sampled from the 54.6 h of data annotated from 22 participants in the Annotated dataset. Sampling from such a large varied annotated dataset could increase model performance relative to sampling from smaller datasets drawn from fewer participants.

### 6.7. Future validation efforts

An important next step of this work is to assess the validity of our auditory chaos model for predicting child behavior and functioning. Given the practical differences between subjective parent reports of chaos and our objective real-time measure we may not expect to see strong correlations between these two measurements. However, these measures could provide complimentary insights into child functioning. At the real-time timescale, we have shown in preliminary work that our chaos predictions correspond to real-time increases in infant heart rate (85), as predicted by previous works that increases in volume leads to increases in infant arousal (15, 36). Future efforts could also examine other real-time indicators, including, e.g. child focus of attention or child regulation, and how these relations differ according to child temperament. In addition, prospective studies could examine how objective measures of household chaos compare to parent reported measures for predicting children’s longitudinal outcomes, including infant negative emotionality (86), behavioral regulation (87), cognitive outcomes (receptive vocabulary and attention), behavioral outcomes (anxiety/depression and attention problems) and effortful control (2).

## 7. Conclusion

In this paper, we developed a multi-class model for real-world auditory chaos classification. To do so, we collected and annotated a huge corpus of real-world auditory chaos, the first and largest of its kind. Our pioneer effort to classify auditory chaos sets the stage for exciting possibilities in developmental psychology. Once validated, automated fine-grained measures of chaos obtained from our model can provide a novel opportunity to systematically and objectively assess household chaos as an everyday risk factor for child behavioral development in naturalistic settings.

For the engineering community, this work provides a demonstration of model development challenges and solutions in the domain of real-world audio classification. High auditory chaos embodies typical real-world activities or environments insofar that it is highly variable, complex, requiring domain specific knowledge to obtain reliable judgements, and rare, meaning that it requires strategies for filtering large volumes of data to obtain a sizeable training dataset. Our work indicated that annotation of such real-world events can benefit from leveraging existing resources to reduce the total amount of data annotated, thereby, reducing annotation time and efforts.

## Data availability statement

The original contributions presented in the study are included in the article/Supplementary Material, further inquiries can be directed to the corresponding authors. A subset of the Annotated dataset is available in the de Barbaro Chaos at https://homebank.talkbank.org/access/Password/deBarbaroChaos.html. HomeBank membership is required to access the dataset. The trained CNN chaos model is available on Github at https://github.com/dailyactivitylab/AuditoryChaosClassification.

## Ethics statement

The studies involving humans were approved by Institutional Review Board of the University of Texas at Austin (Ethics approval number: 2017-06-0026). The studies were conducted in accordance with the local legislation and institutional requirements. Written informed consent for participation in this study was provided by the participants’ legal guardians/next of kin. Written informed consent was obtained from the individual(s), and minor(s)’ legal guardian/next of kin, for the publication of any potentially identifiable images or data included in this article.

## Footnotes

^1^ https://homebank.talkbank.org/access/Password/deBarbaroChaos.html

^2^ https://github.com/dailyactivitylab/AuditoryChaosClassification
