# Supplementary material for: Auditory chaos classification in real-world environments
Source: Front Digit Health. 2023 Dec 21;5:1261057. doi: 10.3389/fdgth.2023.1261057 (PMC10764466; doi:10.3389/fdgth.2023.1261057)
Supplement: Supplementary file 1 [file Datasheet1.pdf]

Supplementary Material

1 AUDITORY CHAOS ANNOTATION SCHEME

This section details the auditory household chaos annotation scheme that was used to annotate the dataset. All sounds are taken into consideration when labeling an audio segment, even including sounds from the target infant wearing the LENA recorder. Every selected 5 second audio segment is labelled with a chaos level from four levels: no (0), low (1), medium (2) or high (3).

1.1 No Chaos (0)

Segments that do not contain any sound (expect for the microphone hum).

1.2 Low Chaos (1)

Segments that contain soft daily sounds are included in this category.

Respiratory sounds. Infant regular or deep breathing sounds. Mild to medium snoring or wheezing or blowing raspberries.

Adult speech. Single person speech or contingent or non-overlapping conversation between individuals (can have one voice in the background and one in the foreground) or two non-contingent non-overlapping speech or in-person singing. Speech or singing should occur at a normal volume level. Includes humming and kissing sounds.

Child speech. Takes into consideration all children in the household i.e. infant wearing the LENA recorder and their siblings. Includes low-volume or background or normal pitch (not attention-grabbing) child speech or singing or infant vocalizations. Includes all neutral infant vocalizations. Includes very distant, isolated, not attention-grabbing child or infant distress.

Music. Low-volume calming music or wind chimes. Includes non-stimulating toy music or sounds. Includes isolated and distant (barely audible/not distinct) bell sound.

Utility sounds. Low-volume dishwasher, washing machine, microwave hum, fan noise. Includes sounds similar to white noise machine, sleep machines, wave machines (need to be calming or consistent/monotone sounds) irrespective of their volume as they are typically placed closed to the infant wearing the LENA sensor. Includes water spraying sounds from a spray bottle, water draining, trickling, dripping, pouring.

Outdoor sounds. Footstep sounds, stroller on pavements or roads, walking on gravel, wind blowing or light breeze, low hum when inside the car.

Impact/Source-ambiguous/Other sounds. Less than or about 2.5 seconds of continuous, non-jarring, everyday impact sounds or less than 4 distinct impact sounds. Includes impact sounds caused by another child playing quietly in the background. Includes sounds, irrespective of volume, from infant clothes rustling, uncovering plastic wrappers, phone vibrating, feet shuffling, soft tapping or patting or sounds caused by moving the LENA sensor.

1.3 Medium Chaos (2)

Segments that contain stimulating sounds are included in this category.

Respiratory sounds. Really loud snoring, coughing loudly and repeatedly, sneezing repeatedly. Blowing raspberries on a surface.

Adult speech. Two non-contingent overlapping speeches or one overlapping conversation (can be one in the background and one in the foreground). Includes harsh, high pitch or raised voices, commanding tones (no shouting), loud in-person singing. Includes adult crying or sudden abrupt loud vocal sounds e.g. - grunts or groans.

Child speech. Isolated infant and child attention-grabbing vocalizations or distress, hiccups between cries. Includes infant laughing or happy loud sound (not squeals), more affective or expressive tones and sounds than neutral sounds. High-pitched or annoying child speech or singing or vocalizations. Includes a child playing - has to be rough, running, a bit stimulating/chaotic.

Electronic speech. Low-volume to normal speech, sounds, singing from TV, radio, phone, speakers. Has to be "recorded" voice through an electronic device.

Music. Stimulating children's music from a toy or electronic device. Includes loud, disjointed, isolated, non-continuous toy sounds or noises. Includes stimulating instrumental music e.g. pop music, continuous bell sounds, rattle, low-volume opera or choir singing.

Utility sounds. Vacuuming or blow drying, elevator or microwave dings, microwave door opening and closing, dishes clanking. Includes more than 2.5 seconds of running water or shower or faucet or dishwasher spray or garbage disposal sounds (has to be loud).

Animal sounds. Isolated, non-continuous or distant dog barking. Birds chirping and cat meowing.

Outdoor sounds. Vehicles passing-by when outside (not loud). Car seat belt notification sound. Distant police siren, emergency vehicle or horn. Low to medium volume/distant public crowded areas (e.g. restaurants, grocery stores, playgrounds, etc.). Low to medium volume chatter in the background (not attention-grabbing, loud or chaotic).

Impact/Source-ambiguous/Other sounds. More than 2.5 seconds of continuous loud impact sounds or 4 or more distinct loud impact sounds. Includes snapping, knocking, clapping or loud infant patting, phone notification sounds. Construction sounds or vehicle sounds heard from inside the house (similar to being muffled).

1.4 High Chaos (3)

Segments that contain highly stimulating or scary/jarring sounds are included in this category.

Adult speech. Adults arguing loudly or shouting. Multiple non-contingent, overlapping conversations similar to that of a chatter.

Child speech. High volume infant or child crying/wailing or tantrumming. Includes screaming, shrieking, squealing (high-pitched), foreground shouting.

Electronic sounds. High volume/loud TV, radio, speakers, phone (does not include phone calls unless people are shouting/screaming/arguing). Must include speech (clearly audible words) and other sources of sounds e.g. exciting music, loud base/beats, loud instruments, applause, orchestra, choir, opera, death metal music, laughter, shouting, cheering, commotion, gun shots, war cries or multiple overlapping speech.

Music. Loud stimulating instrumental music e.g. drums, trumpets, orchestra, bass, high-hat.

Utility sounds. Blender or similar.

Animal sounds. Dog barking loudly and repeatedly, loud crow cawing nearby or other loud bird noises.

Outdoor sounds. Cars honking, close-by traffic or vehicle sounds if you are outside, police or emergency vehicle siren. Public environments like restaurants, schools, playgrounds, public swimming pools, etc. Loud construction sounds, lawn-mover.

Impact/Source-ambiguous/Other sounds. Fire or smoke alarms. Includes loud scary-sounding/jarring impact sounds or loud abrupt sounds or crashes. Can be one sound or multiple.

2 COMPARING MODELS USING PAIRED T-TEST

In order to ascertain the best-performing model, we conducted paired t-test to compare each pair of model performance for the Annotated dataset, Filtered set and Unfiltere set. Average participant-specific weighted F1 score, precision and recall along with their standard deviations for every model for each test set are summarized in Table S1.

2.1 Model Performance on Annotated Dataset

When the models were tested on the 22 participants in the Annotated dataset in a LOPO-CV fashion, the CNN model achieved the highest average weighted F1 score, followed by RF-53f. However, when the weighted F1 scores were compared using paired t-tests, these two models were not statistically differentiated from one another ($t(21) = -0.576, p = 0.571$). The baseline model, RF-3f, had statistically significantly worse performance than the CNN ($t(21) = -8.06, p < .001$) and the RF-53f ($t(21) = -9.77, p < .001$) with reference to average weighted F1 score.

2.2 Model Performance on Filtered Set

When the models were tested on all 21 participants in the Filtered set in a LOPO-CV fashion, the pattern of results mirrored results on the Annotated dataset. The CNN had the highest but statistically indistinct average weighted F1 score from the RF-53f model ($t(20) = -0.377, p = 0.710$) both of which performed substantially and significantly better than the baseline model, RF-3f (CNN: $t(20) = -6.11, p < .001$; RF-53f: $t(20) = -7.24, p < .001$) in terms of average weighted F1 score.

2.3 Model Performance on Unfiltered Set

When the models were tested on all 6 participants in the Unfiltered set in a LOPO-CV fashion, the CNN had the highest performance in terms of average weighted F1 score which was statistically similar to that of the RF-53f model ($t(5) = -0.193, p = 0.854$). The baseline model, RF-3f, had the lowest performance with respect to average weighted F1 score which was substantially and significantly worse than the CNN ($t(5) = -7.52, p < .001$) and the RF-53f model ($t(5) = -9.21, p < .001$)

3 SUPPLEMENTARY TABLES AND FIGURES

3.1 Figures

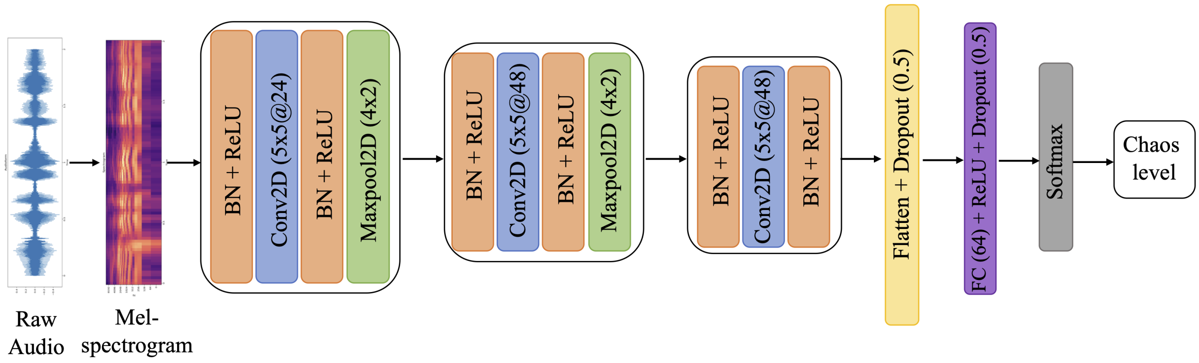

Figure S1. Architecture of our auditory chaos CNN model

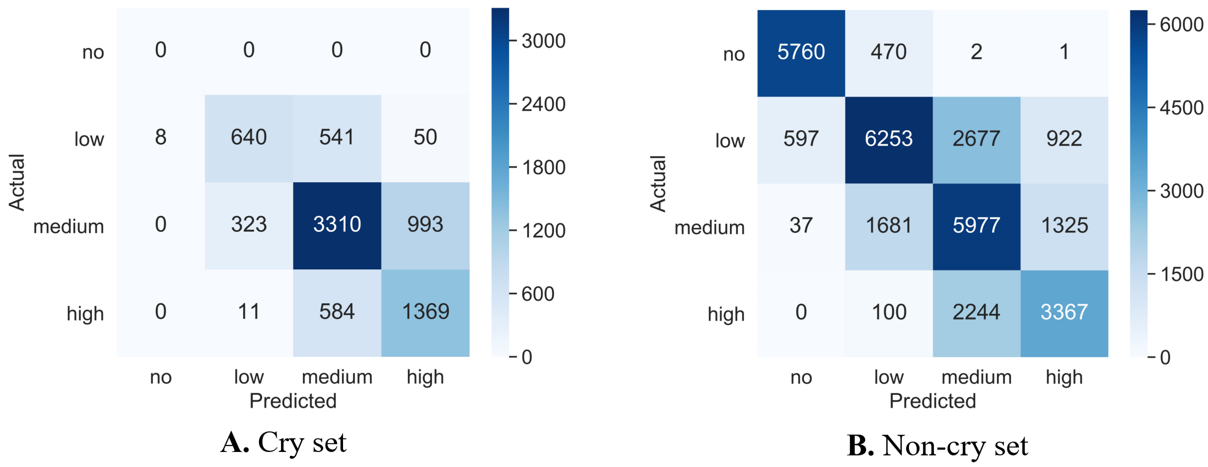

Figure S2. Confusion matrices for the **(A)** Cry set and **(B)** Non-cry set

3.2 Tables

Table S1. Participant-specific weighted model performance for our three models on different test sets

Models	Test Data	F1	Precision	Recall
RF-3f	Annotated (Unfilt. + Filt.)	0.358 (±0.148)	0.558 (±0.226)	0.303 (±0.114)
	Filtered (DS + HS)	0.371 (±0.158)	0.623 (±0.256)	0.305 (±0.122)
	Unfiltered (CA + RS)	0.383 (±0.124)	0.546 (±0.152)	0.323 (±0.103)
RF-53f	Annotated (Unfilt. + Filt.)	0.677 (±0.201)	0.740 (±0.176)	0.672 (±0.196)
	Filtered (DS + HS)	0.680 (±0.259)	0.792 (±0.176)	0.671 (±0.258)
	Unfiltered (CA + RS)	0.679 (±0.128)	0.698 (±0.159)	0.689 (±0.110)
CNN	Annotated (Unfilt. + Filt.)	**0.700** (±**0.160**)	0.770 (±0.155)	0.674 (±0.161)
	Filtered (DS + HS)	**0.705 (±0.189)**	0.805 (±0.169)	0.670 (±0.201)
	Unfiltered (CA + RS)	**0.684 (±0.087)**	0.742 (±0.090)	0.659 (±0.090)

Note: Models were trained using 40 hours of balanced data across four levels of auditory chaos randomly sampled from the Annotated dataset and tested using LOPO-CV on their respective test sets. Participant-specific weighted F1 score, precision and recall were computed for each fold of LOPO-CV and average weighted metrics were calculated by averaging across the results of all 22 participants. Results for each analysis are separated using emphasis lines. Model performances in bold represent the highest average weighted F1 score achieved across all five models for that particular test set.

Table S2. Results of our training data ablation study - Effect of exponentially decreasing amounts of balanced training sets on model performance. All models were trained and tested using LOPO-CV on the Annotated dataset across all 22 participants

Train on Annotated Dataset (Unfilt. + Filt.)	Test on Annotated Dataset (Unfilt. + Filt.)					
Balanced Data (hrs)	Macro			Weighted		
	F1	Precision	Recall	F1	Precision	Recall
40	0.701	0.705	0.702	0.679	0.685	0.680
20	0.670	0.679	0.670	0.650	0.660	0.649
10	0.672	0.689	0.664	0.652	0.665	0.651
5	0.674	0.685	0.674	0.649	0.661	0.651
