# Supplementary figures and images for: Auditory chaos classification in real-world environments
Source: Front Digit Health. 2023 Dec 21;5:1261057. doi: 10.3389/fdgth.2023.1261057 (PMC10764466; doi:10.3389/fdgth.2023.1261057)

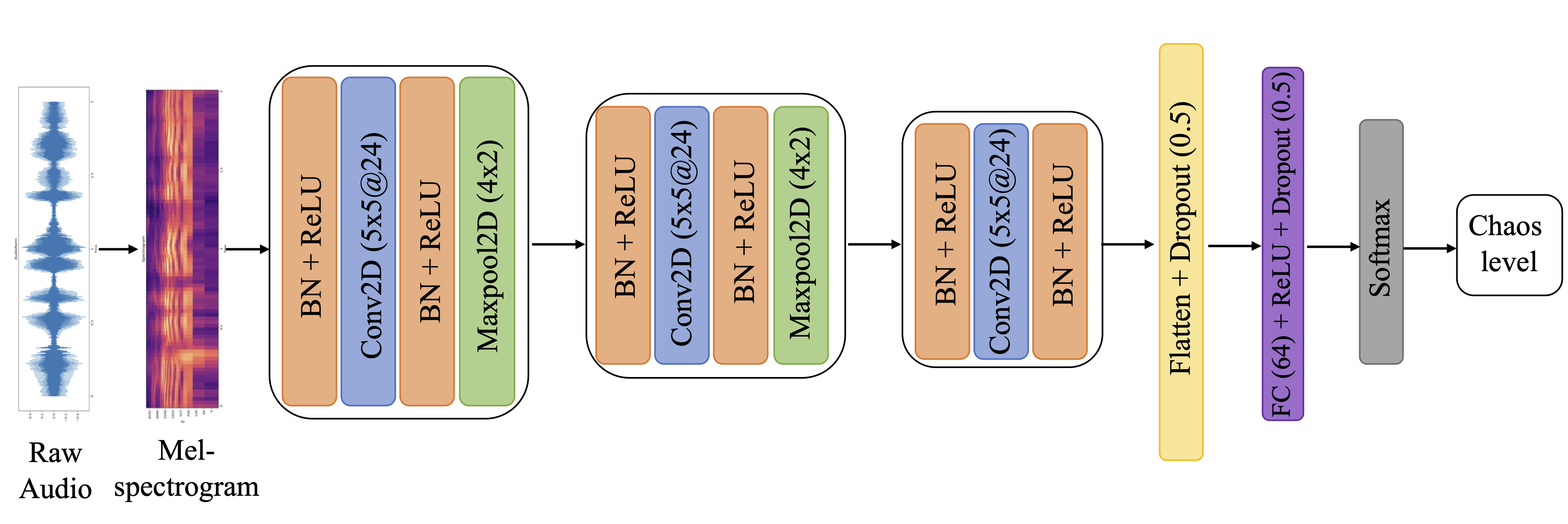

Supplement: Supplementary file 2 [file Image1.tiff]

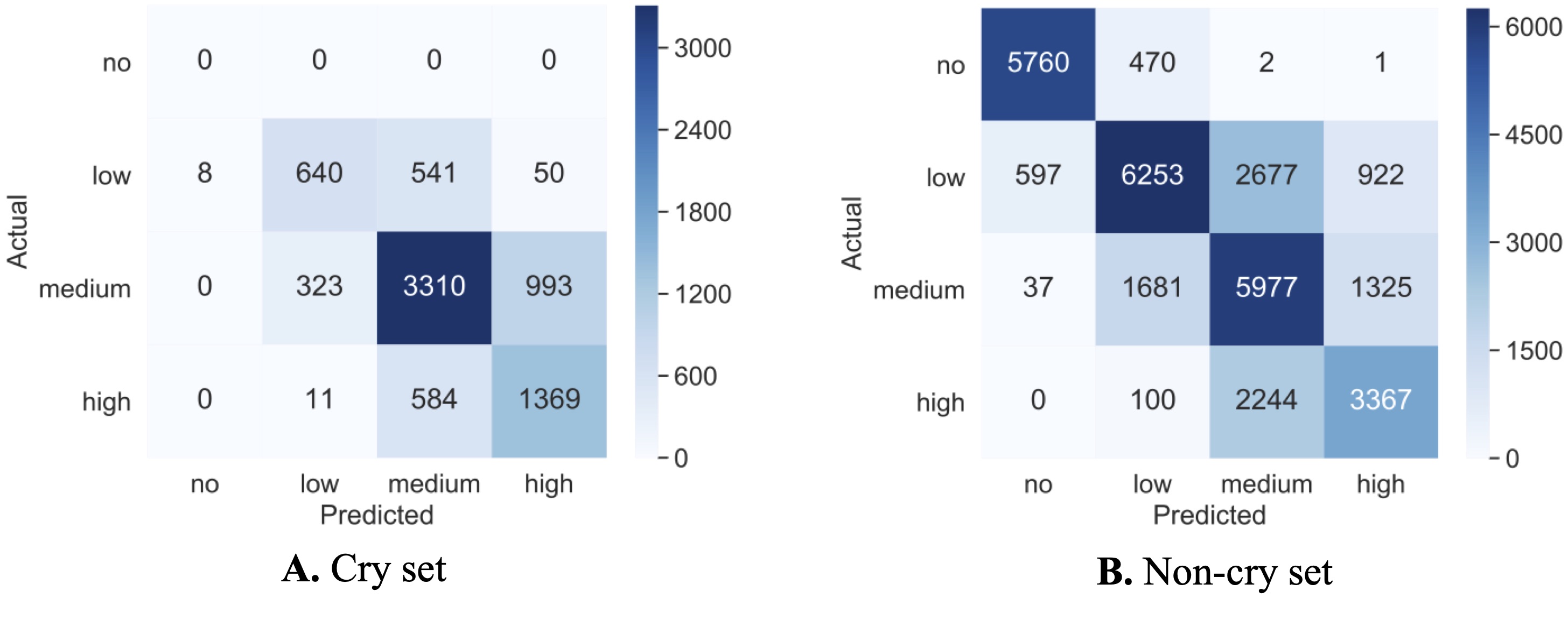

Supplement: Supplementary file 3 [file Image2.jpeg]
